# A mixed-methods study on the design of Artificial Intelligence and data science-based strategies to inform public health responses to COVID-19 in different local health ecosystems: A study protocol for COLEV

**DOI:** 10.12688/f1000research.110958.1

**Published:** 2022-06-22

**Authors:** Catalina González-Uribe, Nicolás Yañez, Alf Onshuus Niño, Nubia Velasco, Juan Manuel Cordovez, Mauricio Santos-Vega, Natalia Niño-Machado, Andres Burbano, Angus Forbes, Ciro Alberto Amaya Guio, Simon Turner, Diana Higuera-Mendieta, Sandra Martínez-Cabezas

**Affiliations:** 1 School of Medicine, Universidad de los Andes, Bogotá, 111711, Colombia; 2 Department of Mathematics, Universidad de Los Andes, Bogotá, 111711, Colombia; 3 Management School, Universidad de los Andes, Bogotá, 111711, Colombia; 4 Department of Biomedical Engineering, Computational & Mathematical Biology, Universidad de los Andes, Bogotá, 111711, Colombia; 5 Department of Architecture and Design, Universidad de los Andes, Bogotá, 111711, Colombia; 6 Department of Computational Media, University of California, Santa Cruz, Santa Cruz, California, USA; 7 Industrial Engineering Department, Universidad de los Andes, Bogotá, 111711, Colombia

**Keywords:** COVID-19, artificial intelligence, decision-making, data science

## Abstract

**Background:** Artificial Intelligence (AI) and data science research are promising tools to better inform public policy and public health responses, promoting automation and affordability. During the COVID-19 pandemic, AI has been an aid to forecast outbreak spread globally. The overall aim of the study is to contribute to the ongoing public health, socioeconomic, and communication challenges caused by COVID-19.

**Protocol:** COLEV is a five-pronged interdisciplinary mixed methods project based on AI and data science from an inclusive perspective of age and gender to develop, implement, and communicate useful evidence for COVID-19-related response and recovery in Colombia. The first objective is identification of stakeholders’ preferences, needs, and their use of AI and data science relative to other forms of evidence. The second objective will develop locally relevant mathematical models that will shed light on the possible impact, trajectories, geographical spread, and uncertainties of disease progression as well as risk assessment. The third objective focuses on estimating the effect of COVID-19 on other diseases, gender disparities and health system saturation. The fourth objective aims to analyze popular social networks to identify health-related trending interest and users that act as ‘super spreaders’ for information and misinformation. Finally, the fifth objective, aims at designing disruptive cross-media communication strategies to confront mis- and dis-information around COVID-19. To understand stakeholders’ perspectives, we will use semi-structured interviews and ethnographic work. Daily cases and deaths of COVID-19 reported from the National Surveillance System (INS) of Colombia will be used for quantitative analysis, and data regarding the online conversation will be obtained from Facebook and Twitter.

**Conclusions:** COLEV intends to facilitate the dialogue between academia and health policymakers. The results of COLEV will inform on the responsible, safe and ethical use of AI and data science for decision-making in the context of sanitary emergencies in deeply unequal settings.

## Introduction

The coronavirus disease of 2019 (COVID-19) has been responsible for more than three million deaths worldwide up to July 2021. ^1^ Latin America is home to approximately 30% of such deaths according to the World Health Organization (WHO). Global vaccine research and development against COVID-19 was unprecedented in terms of scale and speed ^1^; in May 2020, there were 73 candidates in the pre-clinical stages. On December 2020, 13 of the vaccine candidates were on phase 3, and by February 2021, four vaccines were already licensed for emergency use in several countries. ^1^ Likewise, academic cooperation and production increased at rates never seen before, ^2^ evidenced by an increase of 92% in submissions of health and medicine manuscripts during 2020 compared to 2019. ^3^

Artificial Intelligence (AI) and data science research are promising tools to better inform public policy and public health responses, promoting automation and affordability. ^4^ During the COVID-19 pandemic, AI aids to forecast outbreak spread globally. ^5^^–^^7^ Other AI applications have been documented on clinical applications aiding diagnosis and prognosis about COVID-19, ^8^ and contact tracing and strict enforcement of quarantines. ^9^ However, with the recent advances and applications in the field of AI to tackle COVID-19, ethical tensions have risen. ^10^ The implementation of AI solutions might reinforce bias and discrimination, thus exacerbating social inequalities. ^11^^,^^12^ For instance, when discriminatory structures are ingrained in datasets used to train algorithms, the AI systems will be biased and will reproduce, and even reinforce, the already discriminatory practices. ^12^ In this context, diverse stakeholders and developer groups are essential to avoid unintended harmful consequences of building AI systems from a dominant, “one fits all” point of view.

With the pandemic, pre-existing social inequalities became more evident worldwide. The effect of the pandemic with regards to deepening social disparities are expected to be the highest in Latin America, where income inequality is the highest in the world; the top 1% wealthiest individuals owned 24.9% of the total income for 2019, while in the world, the same proportion of individuals owned the 19.1% of the income. ^13^ Colombia, is no exception since it is one of the countries with the highest income inequality in the region and this situation is exacerbated by having also one of the highest labor market informality in Latin America, according to The World Bank In Colombia. Particularly, during 2020, 3.4 million people became unemployed, but women were disproportionally affected; while there was an 18% reduction in male employment, there was a 27.2% reduction for women. ^14^ Additionally, there was an increase of 123% in emergency calls for domestic violence evidencing how gender disparities intensified. ^15^

Social networks and mass media have played a fundamental role in disseminating public health content in recent years. ^16^ The COVID-19 pandemic increased the public exposure to information regarding the prevention and management of the diseases. ^16^ With massive amounts of information online, there is increased exposure to hoaxes and misinformation as well. ^17^ Research has shown that misinformation is correlated with engaging in erroneous health practices that increase the spread of COVID-19 and leave room for several conspiracy theories and mistrust in health authorities and professionals. ^17^^,^^18^

This protocol describes a five-pronged interdisciplinary research endeavor based on AI methods and data science from an inclusive perspective of age and gender to develop, implement, and communicate useful evidence for COVID-19-related response and recovery in Colombia, South America. The overall aim of the COLEV study is to contribute to the ongoing public health, socioeconomic, and communication challenges caused by the COVID-19 pandemic, as part of an initiative of the International Development Research Centre (IDRC) of Canada and the Swedish International Development Cooperation Agency (Sida) aiming to understand the response to the COVID-19 pandemic and support research based on AI in low and middle-income countries.

## Protocol

### Study design

COLEV is a mixed-methods study on the design of AI and data science-based solutions to inform public health responses to COVID-19 in different local health ecosystems. Our purpose is to produce and communicate evidence for differential public health measures to address COVID-19-related challenges in Colombia tailored to regional contexts and vulnerable populations, through the interdisciplinary rigorous ethical use of AI and data science. ^19^

We have five principal aims: 1) To identify health ecosystem stakeholders’ preferences and perceived needs regarding AI and data science, and their use in decision-making relative to other forms of evidence in response to COVID-19. 2) To develop a long-term and real-time model that allows forecasting of COVID-19 cases, hospital and intensive care unit (ICU) occupancy, and COVID-19 fatalities, based on estimated epidemiological parameters that account for age, socioeconomic, gender, and comorbidity variation and tailored input for decision making allowing to compare the impact of possible interventions and targeting resources. 3) To evaluate the impact of COVID-19 on health and health-related outcomes and their effect on the resource management policies for the health ecosystems. 4) To characterize the online public conversation about COVID-19 analyzing popular social networks with AI methods. And 5) to design a disruptive communication strategy tailored to different societal groups to confront mis- and dis-information around COVID-19 and reinforce our local and national public health measures and policies. Table 1 summarizes the research questions that we seek to answer.

**Table 1.** Research question and data sources according to aims of the study.

Aim	Research questions	Type of data collection	Data	Source
**1**	1.1 What are decision-makers' preferences and perceived needs for AI and data science in response to COVID-19, taking into account technical, ethical, and social aspects of its use?	Primary	Preferences and perceived needs of Stakeholders	Semi-structured interviews and ethnographic work
	1.2. What are the barriers and facilitators to the use of data science and AI in health system decision-making on COVID-19, in different local health system contexts and applications?	Primary	Barriers and facilitators of the use of data science and AI	Semi-structured interviews and ethnographic work
**2**	2.1. What are the possible trajectories of the disease in real-time and the uncertainty associated with every trajectory?	Secondary	-Cases of COVID-19 in Colombia -Deaths due to COVID-19 in Colombia	National Epidemiologic Surveillance System from the National Institute of Health: ‘ *Instituto Nacional de Salud*’
	2.2. What are the factors that modulate space-time variation in COVID-19 infections in different regions of Colombia?		-Cases of COVID-19 in Colombia at a departmental level -Deaths due to COVID-19 in Colombia at a departmental level	National Epidemiologic Surveillance System from the National Institute of Health: ‘ *Instituto Nacional de Salud*’
**3**	3.1. What are the effects of COVID-19 on chronic, and mental health outcomes?	Secondary	-Number of reported healthcare visits due to coronary outcomes, and mental health disorders.	National Record of Services from the Ministry of Health: ‘ *Ministerio de Salud y Protección Social*’
	3.2. What are the effects of COVID-19 on gender disparities, vulnerable populations, and regions?	Secondary	-Labor force, income, quality of life in Colombia disaggregated by gender and departments	National Household Surveys: ‘ *Departamento Nacional de Estadísticas*’
	3.3. What is the impact in sexual, gender-based and domestic violence, and adolescent pregnancies?	Secondary	-Annual reports of domestic violence in Colombia	National Forensic Office: ‘ *Instituto de Medicina Legal*’
			-Annual reports of sexual felonies	National Forensic Office: ‘ *Instituto de Medicina Legal*’
			-Annual records of life births in Colombia	Vital Statistics from the National Statistics Department: ‘ *Departamento Nacional de Estadísticas*’
	3.4. How to improve resource management in the health ecosystems incorporating different efficiency criteria?	Secondary	-Daily report of applied doses of vaccines against COVID-19 per manufacturer at a departmental level	National Record of Vaccination from the Ministry of Health: ‘ *Ministerio de Salud y Protección Social*’
**4**	4.1. What are the health-related trending or viral topics of interest for vulnerable groups or populations?	Secondary	-Facebook and Twitter COVID-19 data streams -The Americas Barometer Survey	-Facebook -Twitter -‘ *Observatorio de la Democracia*’
	4.2. What are the prevailing reactions to policies and events of social importance related to COVID-19 in Colombia?			
	4.3. Who are the key users, profiles, or accounts that act as 'super spreaders' for information, dis-information, and misinformation diffusion?			
**5**	5.1. What are the key variables in data related to mis- and dis-information around COVID-19 to gain insights to take action implementing a dynamic communication process?	Secondary	-Curated pieces of mis- and dis-information associated with COVID-19 in Colombia	-National network of fact checkers: Colombiacheck
	5.2. How to tailor the strategy to combat mis- and dis-information about COVID-19 considering specific societal groups including vulnerable communities?	Primary	-Perceptions of media outlets regarding the structure of the message delivered using Data-Driven Journalism and Data Visualization Storytelling	-Workshops with media outlets

COVID-19, coronavirus disease of 2019; AI, Artificial Intelligence.

Ethical approval has been obtained by Ethics Committee at the Universidad de Los Andes (Acta: No.1394 – 2021). The participants will provide written informed consent to participate in this study.

### Study setting

Colombia is located in South America, with a population of 50,372,424 with about two million indigenous and three million Afro-Colombians, ^20^ and has profound social inequalities as evidenced by its income Gini coefficient (0.51 in 2019), ^21^ and deep urban/rural disparities. For example, the multidimensional poverty index was 17.5% at national level in 2019, while in rural areas was 34.5%. ^22^

The Colombian health system is an insurance-based model with 98% of the population affiliated. Amid social inequalities, the healthcare system is based on the solidarity principle in which employees and self-employed workers with capacity to pay, along with taxes, cover the affiliation of those of lower income and unemployed who account for 47% of the population. ^23^

During 2020, Colombia implemented different measures to mitigate and control the COVID-19 pandemic, land and river borders were closed, schools and universities were closed, and a mandatory lockdown was declared on March 24/2020, which lasted until August 31/2020. In September, a selective lockdown phase began to mitigate the economic impact of the virus locally and to allow productive life. ^24^

### Data collection

This study employs a mixed-methods design. The quantitative and qualitative data will be obtained from several sources, mentioned below:

*Qualitative data collection*

To understand stakeholders’ perspectives on the development and use of AI and data science for public health and COVID-19 responses in the country, we will use semi-structured interviews and ethnographic work. Interviews will be conducted with stakeholders of relevance in decision-making about the COVID-19 response, and ethnography will be conducted with scientists or engineers developing technologies based on AI and data science. We will observe the processes of developing technological tools and their efforts to transfer them to the field of policy. Stakeholders include national and local government representatives, health policy planners, health providers, research centers, and AI and data science developers and experts. We will develop a map of actors to select potential interviewees. We will leverage our current, close and longstanding relationships with such stakeholders to recruit our participants. Then, we will follow a snowball sampling until we reach theoretical saturation. In addition to the stakeholders’ interviews, we will select relevant case studies, up to three, of AI development or use targeting COVID-19 that we can analyze using ethnography. All interviews will be audio-recorded and transcribed. The interviews, fieldwork diaries, and relevant documents will be coded and analyzed using NVivo (NVivo, RRID:SCR_014802).

Additionally, data from curated pieces of mis- and dis-information associated with COVID-19 in Colombia should be collected from the national network of factcheckers, Colombiacheck. The number of pieces of mis- and dis-information will depend on the criteria that Colombiacheck uses to publish and check including virality, and engagement.

*Quantitative data collection*

Quantitative data will rely on secondary sources. We will use daily cases and deaths of COVID-19 reported from the National Surveillance System ‘ *Instituto Nacional de Salud*’ ( INS) of Colombia. The data from other morbidities, such as mental health disorders, will be extracted from the National Record of Services reported by the Ministry of Health. ^25^ Mortality due to other causes different from COVID-19, live-births by ages and sex at the municipal level will be taken from the Vital Statistics generated by the Department of National Statistics of Colombia (DANE). The population data will be extracted from the population projections calculated by DANE. And, the information of daily doses of vaccines is available from the official records and reports of the Ministry of Health.

Data regarding the online conversation will be obtained from Facebook, and Twitter COVID-19 data streams. These data sources will be complemented with The Americas Barometer survey, conducted by ‘ *Observatorio de la Democracia*’ (Democracy Observatory). Table 1 summarizes the pool of secondary sources according to aims and research questions.

The interview guides that will be used to collect data can be found as *Extended data.* ^38^

### Analytical approach

To answer the research questions for each objective, an interdisciplinary team will be formed including researchers from several disciplines such as data scientists, social scientists, and health professionals. An advisory committee with national and international experts in public health and governance was created to guide the project’s priorities, analyze, and validate the results. To establish a common language and to deliver aligned products, the team is going to work around four cross cutting topics: return to schools, vaccination, mental health, and vulnerable groups such as migrant populations.

**Figure 1.**
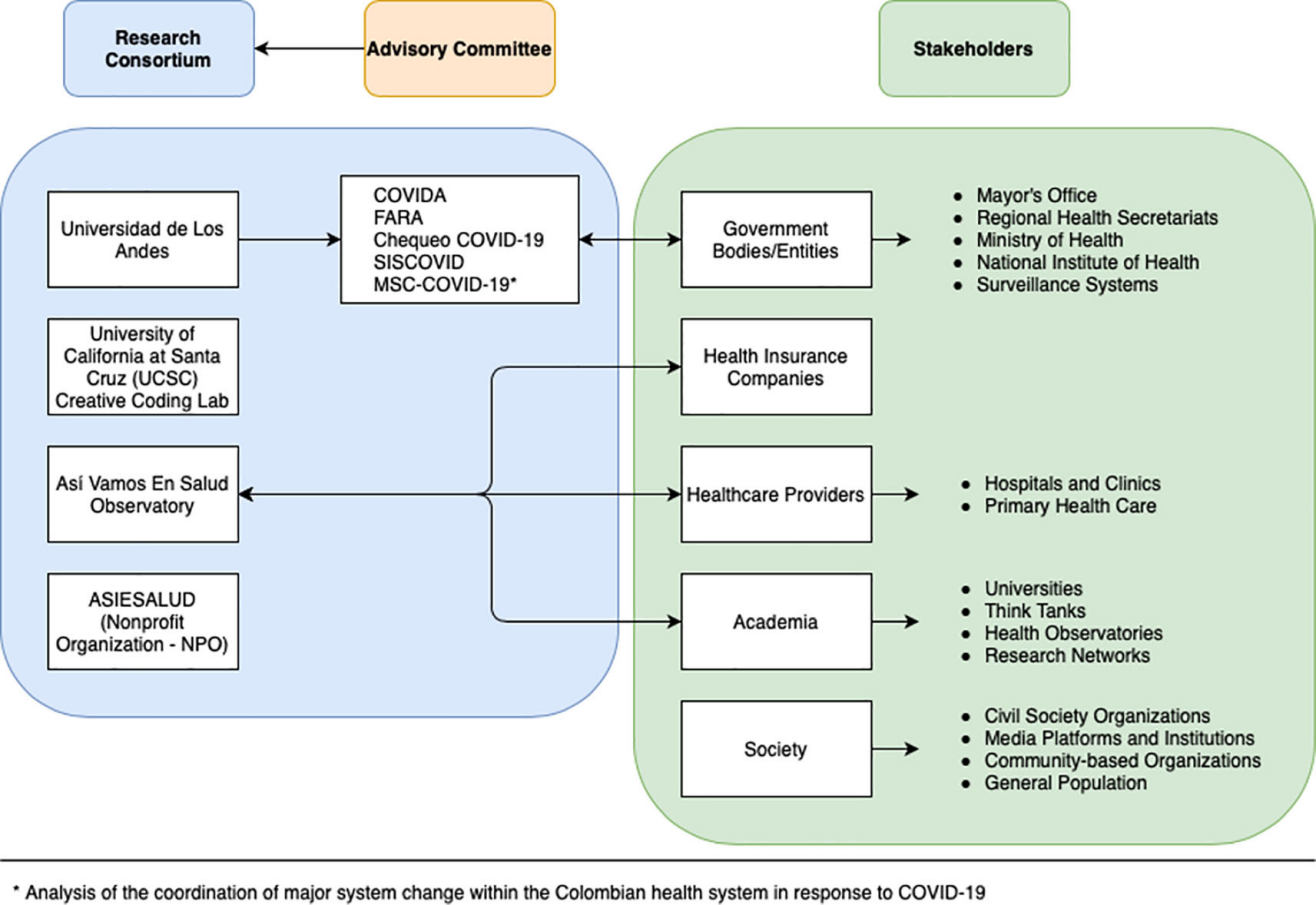
Governance framework to be used in the implementation of COLEV. COVID-19, coronavirus disease of 2019.

COLEV was envisioned as a means to co-construct AI and data science-based solutions to inform public health responses to COVID-19 in different local health ecosystems. It builds upon the experience of several research groups from Universidad de los Andes, University of California–Santa Cruz (UCSC) and Non-Governmental Organizations (NGOs) like the health observatory ‘ *Así Vamos en Salud*’ and ‘ *ASI ES SALUD*’. These two organizations are articulated and plan to use COLEV results to guide actions in the context of the pandemic leveraging on long standing relationship with stakeholders in Colombia ( *i.e.*, decision makers, other academic groups, the civil society, and health care providers and ensures). We recognize the need for collaboration between different institutions and research projects, interacting with different sectors such as education, health, city planning, amongst others ( Figure 1).

The analysis plan for each objective is described below:

**Objective 1. To identify health ecosystem stakeholders’ preferences and perceived needs regarding AI and data science, and their use in decision-making, relative to other forms of evidence in response to COVID-19.** Upon recording and transcription of the interviews, we will conduct a thematic analysis ^26^ that will be centered in comparing processes of development, communication, and use of data to inform decision-making on COVID-19. The ethnography will involve tracing how AI and data science are used for different applications in relation to COVID-19 so that comparisons can be drawn between data use in different geographical localities and a variety of applications in public health. We will conduct joint mapping of stakeholder arrangements, informing the topics discussed in interviews, and joint observations of decision-making processes.

**Objective 2. To develop a long-term and real-time model that allows for forecasting of COVID-19 cases, hospital and ICU occupancy, and COVID-19 fatalities, based on estimated epidemiological parameters that account for age, socioeconomic, gender, and comorbidity variation and tailored input for decision-making allowing to compare the impact of possible interventions and targeting resources.** We will start by developing and providing a forecasting system for the country that combines space-time AI approaches, statistical inference methods, and data assimilation algorithms. We will combine different forecasting approaches to create a standardized set of data on forecasts making projections of COVID-19 cases, hospital and ICU occupancy, and COVID-19-fatalities in Colombia. Multiple outputs from the model will be collected, standardized, visualized, and synthesized in a dashboard with accuracy measures and description of the methods. Every week, we will update our COVID-19 Forecast ensemble and interactive visualization using the most recent forecast from each approach.

**Objective 3. To evaluate the impact of COVID-19 on health and health-related outcomes and their effect on the resource management policies for the health ecosystems.** We will gather secondary data regarding non-communicable diseases ( *e.g.*, cardiovascular diseases), mental illnesses ( *e.g.*, depression and anxiety); adolescent pregnancies, domestic violence, and other infectious diseases ( *e.g.*, Dengue, Malaria). We will develop descriptive and predictive models using data mining, ^27^ identifying trends and behaviors before and after the arrival of COVID-19.

To characterize the process services, we will describe the pathway followed by a patient in the system and the resources consumed. To do that, we will use time-motion studies and process and network analysis. The time-motion studies are useful for identifying the stages, times, physical and technical resources, and staff required to provide a specific health service. ^28^^,^^29^

**Objective 4. To characterize the public online conversation about COVID-19 analyzing popular social networks with AI methods.** This study will use the Twitter data stream starting from March 1, 2020, based on the keywords “COVID-19”, “coronavirus”, and “Colombia”. Foundational information ( *i.e.*, raw text, user IDs, timestamps, unique tweet ID, among others), sub dictionaries ( *i.e.*, user, place, extended tweets, retweeted status), as well as any other available metadata ( *e.g.*, language, retweets, favorites, replies), will be retrieved.

We will organize the information as Pandas DataFrame, and any hierarchical structures will be flattened. The Twitter data stream will be analyzed in two ways: 1) as snapshots of periods of time based on the occurrence of key events of national importance and peaks in Twitter usage and content creation, and 2) as a time series with the intent of capturing content variation over time. Sentiment analysis is a useful technique to indicate the prevailing emotion attached to a specific 28/59 keyword, cluster, or network employing several machine learning algorithms of the natural language processing (NLP) family. To this end, the VADER toolkit of the NLKT python package will be used for tweets and replies. This toolkit allows for the interpretation of capital letters, exclamation marks, and emojis as well as raw text. A pilot analysis of a 1% sample of the retrieved tweets will be assessed manually in order to confirm the face validity of the algorithm. The same procedure will be repeated using Facebook data.

**Objective 5. To design a disruptive communication strategy tailored for different societal groups to confront mis- and dis-information around COVID-19 and our local and national public health measures and policies.** We will follow a three-step routine:

First, we will develop custom-made solutions to organize, systematize, and visually display the data acquired to identify and represent patterns and insights regarding the narrative components of COVID-19 mis- and dis-information in the Colombian context. ^30^^,^^31^ Several of the tools that will be used are based on JavaScript and Python integration with AI and ML methods, such as Text Mining, Statistical Natural Language Process (NLP), and NLP topic modeling, which are previously applied to translate into rich visual experiences of high accuracy that help with information understanding in the decision-making process. ^32^

Second, after having a visual representation of the data, we will share this visualizations with the public sector media, traditional media leaders, and digital media key practitioners interested in the problem of mis- and dis-information around COVID-19 using User-Centered Design and Service Design methodologies. ^33^^,^^34^ We will conduct workshops with these media outlets to discuss the insights about the use of Data-Driven Journalism and Data Visualization Storytelling to communicate them. ^35^^,^^36^

Third, upon identification of the narrative components we will then design and prototype the disruptive communication strategy. We will use Speculative Design and Co-Design methods to break and subvert the narratives created by mis- and dis-information going back to the media stream with concrete interventions. ^37^

### Ethical considerations

This study was approved by the Ethics Committee at Universidad de Los Andes (Acta No.1394 – 2017). All subjects will sign an informed consent form during the qualitative data collection. Findings will be disseminated through open access publications, academic events, newspaper outlets, and presentations with stakeholders.

### Study status

For the quantitative components, we are currently in the data collection stage. Interviews are being conducted, and snowball sampling is still in place. The researchers have not yet reached theoretical saturation. Researchers are currently cleaning and depurating the datasets provided by official sources in the quantitative component. Additionally, the team is currently designing Application Programming Interfaces that facilitate automatic acquisition and cleaning of the datasets to be ready for analysis.

## Discussion

The COLEV study aims to generate evidence for decision-makers at the local level. The interdisciplinary nature of our working group will allow a greater understanding of the complexity of the pandemic in a country in which inequalities have increased. We hope to facilitate the dialogue between the academia and health policymakers by first exploring their needs, priorities and concerns regarding AI and data science solutions for COVID-19 control; and then co-construct such strategies. Additionally, we expect to identify the main super-spreaders of misinformation in social networks and build disruptive communication strategies to combat misinformation with an emphasis on COVID-19 vaccination and the emergent main topics of the online conversation in Colombia.

The results of COLEV will inform local and regional researchers and stakeholders on the responsible, safe and ethical use of AI and data science for decision-making in the context of sanitary emergencies in deeply unequal settings.

## Data availability

### Underlying data

No data are associated with this article.

### Extended data

Open Science Framework: Decisions and Data. https://doi.org/10.17605/OSF.IO/AYU9W. ^38^

This project contains the following extended data:

- Interview guide - Stakeholders.pdf
- Interview guide - Stakeholders involved in COVID-19 decision making processes.pdf
- Participant informed consent.pdf

Data are available under the terms of the Creative Commons Zero “No rights reserved” data waiver (CC0 1.0 Public domain dedication).
