# Supplementary material for: A mixed-methods study on the design of Artificial Intelligence and data science-based strategies to inform public health responses to COVID-19 in different local health ecosystems: A study protocol for COLEV
Source: F1000Res. 2022 Jun 22;11:691. [Version 1] doi: 10.12688/f1000research.110958.1 (PMC11413556; doi:10.12688/f1000research.110958.1)
Supplement: Peer review history

# Reviewer report: Reviewer response for version 1

**DOI:** 10.5256/f1000research.122623.r161479

**Published:** 2024-09-19

**Authors:** Leonardo Arregoces

**Affiliations:** 1 London School of Hygiene and Tropical Medicine, London, WC1E 7HU, UK

Many thanks for considering me for reviewing this manuscript. It is a well thought, very ambitious research protocol that aims to provide relevant information for decision-making at different levels. The researchers do a great job in discussing with decision-makers their information needs and using their answers to develop models and dashboards suitable for informing the complicated decisions that the Covid-19 pandemic brought. Building an interdisciplinary team with skills in qualitative and quantitative skills makes the study robust. It is a quite ambitious project, so I hope the objectives were achieved.

 That being said, it is complicated to review a research protocol after almost three years of pandemic. Because there are no dates on the manuscript, it is hard to tell if the protocol was originally written or planned as stated, or if it had amendments over time. All research suffers changes overtime. It is almost impossible to assess a priori changing circumstances that require adjustments to the protocol. Given that this protocol is being submitted for publication after the work was done, I suggest dates are added and a description of the changes that were needed overtime. This could be at the end of the paper after the analysis plan is described. Probably it could inform better future planning to have a thorough reflection on what was planned at the beginning and what changes were needed over time. It could be called lessons learned or something that capture the flexibility of the team in adjusting to the fast-changing situation that the Covid-19 pandemic showed.

 Although the product of this research is offered to be mostly graphical, such as dashboards and visual results of modelling, the protocol does not use a graphic method to describe how the objectives work together, how the information flows to meet them. For example, objective 1 aims at understanding preferences and information needs in the decision-making process, while objective 4 aims at characterising online conversations and sentiments. It is not clear if that feeds into objectives 2 or 5. A visual aid of how objectives work together to attain a higher purpose of better health would be appreciated. Theory of change might be interesting here.

 Finally, the paper is very well written, easy to read and understand what the research team is aiming for. I believe taking into consideration my two suggestions might be useful for this team and other researchers when planning future endeavors of the magnitude of this work.

Is the study design appropriate for the research question?

Yes

Is the rationale for, and objectives of, the study clearly described?

Yes

Are sufficient details of the methods provided to allow replication by others?

Yes

Are the datasets clearly presented in a useable and accessible format?

Not applicable

Reviewer Expertise:

Health policy, health economy, health financing, pharmaceutical and medical devices policy, regulatory affaris, health technology assessment

I confirm that I have read this submission and believe that I have an appropriate level of expertise to confirm that it is of an acceptable scientific standard, however I have significant reservations, as outlined above.

# Reviewer report: Reviewer response for version 1

**DOI:** 10.5256/f1000research.122623.r173708

**Published:** 2023-09-14

**Authors:** Arriel Benis

**Affiliations:** 1 Digital Medical Technologies, Holon Institute of Technology, Holon, Tel Aviv District, Israel

This manuscript is a research protocol dealing with a project entitled COLEV. COLEV's main and final aim is to deliver disruptive cross-media communication strategies to confront mis- and disinformation around COVID-19 and pandemics. COLEV will be based on AI approaches and data science processes developed with standard tools.

 The protocol is clear and exhaustive.

 However,

1. The authors could more clearly point out (1a) the start and end dates of the qualitative and quantitative data collection, (1b) the expected date of the publication of the results.
2. The authors are not providing clear information about the Machine Learning algorithms they will use and why.
3. It will be interesting to add a short "Discussion" dealing with the
  (3a) "Strengths and Limitations" and 
  (3b) the expected results for operational perspectives (i.e., policies) of this protocol.

Is the study design appropriate for the research question?

Yes

Is the rationale for, and objectives of, the study clearly described?

Yes

Are sufficient details of the methods provided to allow replication by others?

Partly

Are the datasets clearly presented in a useable and accessible format?

Not applicable

Reviewer Expertise:

Health Data Science, Healthcare Informatics, Digital Health, Public Health, Epidemiology, Health Communication, One Health, One Digital Health

I confirm that I have read this submission and believe that I have an appropriate level of expertise to confirm that it is of an acceptable scientific standard, however I have significant reservations, as outlined above.

# Response

**Published:** 2024-04-08

**Authors:** Catalina González-Uribe

**Affiliations:** Universidad de los Andes, Colombia

**Response to reviewers:**

 Thanks you for your comments, below you can find our responses. 

**The authors could more clearly point out: **

- **(1a) the start and end dates of the qualitative and quantitative data collection**

Quantitative data was collected between January 2019 and September 2021, and qualitative data was collected between March 2021 and July 2022.

- **(1b) the expected date of the publication of the results.**

As the aim of our project is to support and inform decision-making amid the COVID-19 pandemic, results will be shared with stakeholders and decision-makers as they are being produced.

**The authors are not providing clear information about the Machine Learning algorithms they will use and why.**

 Data processing and analysis will be done using Python, R, and Spark. We will organize the queried information in data frames, and any hierarchical structures resulting from the original JSON formatting will be flattened.

**It will be interesting to add a short "Discussion" dealing with the: **

- **(3a) "Strengths and Limitations" **

The strengths of this study are its comprehensive coverage of a wide range of topics related to the use of AI and data science in addressing the COVID-19 pandemic in Colombia. The study is interdisciplinary, involving researchers from various fields, including public health, epidemiology, computer science, and social science. Additionally, the study employs mixed-methods, incorporating both qualitative and quantitative data collection methods. Furthermore, the study is specifically tailored to the unique social, economic, and political challenges of Colombia, making it highly relevant to the country's context.

 It's essential to acknowledge that the tools used in this study are still in their initial stages. Therefore, it may be too soon to determine the impact of the AI and data science interventions being developed. Furthermore, it's worth noting that the study focuses specifically on Colombia, and the findings may not apply to other countries.

  

- **(3b) the expected results for operational perspectives (i.e., policies) of this protocol.**

The results of the study will be used to shape public policy and address the needs of decision-makers and stakeholders. The study is expected to have specific applications, such as predicting the number of COVID-19 cases, estimating the effectiveness of interventions like social distancing and quarantine, and supporting policies and decision-making related to the reopening of schools and the logistics of vaccination.

# Reviewer report: Reviewer response for version 1

**DOI:** 10.5256/f1000research.122623.r163597

**Published:** 2023-03-07

**Authors:** Natali Valdez

**Affiliations:** 1 Women's and Gender Studies, Wellesley College, Wellesley, MA, USA

The aim of this project and paper are to co-create data science-based solutions to inform public health responses to pandemics. The research was rigorously and thoughtfully designed to include both qualitative and quantitative approaches that critically inform processes of Big Data. This type of methodological approach is vital to the advancement of precise technological innovations at local and global scales. The strengths of this study protocol are that the  objectives are clearly outlined and feasible, and that the broader impacts of this study are clearly connected to methodological design.

 My understanding is that COLEV is a mixed methods project composed of interdisciplinary scholars that aim to tackle the urgent issue of developing and communicating transparent, trustworthy data for the creation of effective public health interventions on COVID-19. It aims to address this large issue through 5 objectives.

 These objectives center around 

 1) Understanding the ways in which AI and data science are used to communicate topics related to COVID-19, which draws on ethnographic data from people who are using data science to inform decisions about public health interventions.

 2) To develop a forecasting system based on a variety of public health data in relation to COVID-19.

 3) To understand the impact of COVID-19 on related health outcomes and resource management systems, which draws uniquely on a patient-centered perspective.

 4) To analyze public conversations of COVID-19 drawing on available twitter data in order to illustrate the understanding or comprehension among people in Colombia.

 5) To disrupt avenues of disinformation, this objective is closely connected to objective #4 and work together to address the impacts of effective medical/scientific communication.

 All of these objectives are valuable and important in narrating a complex and nuanced understanding of the role of data science in relation to pandemics. The report outlines clear strategies for addressing these objectives with strong methodological research design.

Is the study design appropriate for the research question?

Yes

Is the rationale for, and objectives of, the study clearly described?

Yes

Are sufficient details of the methods provided to allow replication by others?

Yes

Are the datasets clearly presented in a useable and accessible format?

Yes

Reviewer Expertise:

clinical trial design, evidenced-based medicine, public health, epigenetics, maternal health

I confirm that I have read this submission and believe that I have an appropriate level of expertise to confirm that it is of an acceptable scientific standard.
